# Measuring the diversity gap of cannabis clinical trial participants compared to people who report using cannabis

**DOI:** 10.1038/s41598-023-36770-5

**Published:** 2023-06-16

**Authors:** Heather Barkholtz, Maia Bates

**Affiliations:** 1 Forensic Toxicology, Environmental Health Division, Wisconsin State Laboratory of Hygiene, 2601 Agriculture Dr., Madison, WI 53718 USA; 2 Pharmaceutical Sciences, School of Pharmacy, University of Wisconsin-Madison, 777 Highland Ave., Madison, WI 53705 USA; 3 Department of Chemistry, College of Letters of Science, University of Wisconsin-Madison, 1101 University Ave., Madison, WI 53706 USA

**Keywords:** Clinical trials, Epidemiology, Clinical pharmacology

## Abstract

Little is known about the demographics of people who use cannabis, including how use trends within population subgroups have evolved over time. It is therefore challenging to know if the demographics of participants enrolled in cannabis clinical trials are representative of those who use cannabis. To fill this knowledge gap, data from the National Survey on Drug Use and Health (NSDUH) on “past-month” cannabis use across various population subgroups in the United States was examined from 2002 to 2021. The most notable increases in “past-month” cannabis use prevalence occurred in those aged 65 and older (2,066.1%) and 50–64-year-olds (472.4%). In 2021, people reporting “past-month” cannabis use were 56.6% male and 43.4% female. Distribution across self-reported race and ethnicity was 64.1% White, 14.3% Black, 14.1% Hispanic, and 3.1% more than one race. And many ages were represented as 24.4% were 26–34, 24.1% were 35–49, 22.4% were 18–25, and 17.6% were 50–64 years old. To understand if these population subgroups are represented in cannabis clinical trials, participant demographics were extracted from peer-reviewed clinical trials reporting on pharmacokinetic and/or pharmacodynamic models of cannabis or cannabinoids. Literature was grouped by publication year (2000–2014 and 2015–2022) and participant prior exposure to cannabis. Results identified that cannabis clinical trial participants are skewed toward overrepresentation by White males in their 20s and 30s. This represents structural discrimination in the research landscape that perpetuates social and health inequities.

## Introduction

The shifting legal status of cannabis in the United States (US) and internationally is resulting in increased research interest into cannabis and cannabinoid pharmacokinetics and pharmacodynamics^1–4^. Cannabis and cannabinoid pharmacology and epidemiology are the focus of several federal Requests for Proposals and Notices of Special Interest. Despite renewed pharmacological research interest, little is known about the demographics of people who currently or have recently used cannabis, including how use trends have evolved over time. In 2018, an extensive review on national trends in adult cannabis use was published^5^, considering data from 2002 through 2014 in participants aged 18–25 and those aged 26 and older^6,7^. It was noted that increases in cannabis use occurred across sex, region, educational level, and employment status^6,8^. However, the detailed demographics of people who use cannabis was not critically assessed nor reported. In 2019, Cerdá *et. al.* found that recreational cannabis legalization was associated with increases in frequent cannabis use among adults^9^. However, impacts of demographic variables beyond age were not assessed. Also in 2019, Hasin *et. al.* published a narrative review on cannabis use trends by sociodemographic subgroups, summarizing data from several national surveys and a literature review^10^. They concluded that cannabis use had increased across all sociodemographic subgroups including age sex, race, ethnicity, educational level, and location^10^. While this work was comprehensive and complete, survey results through 2015 were considered, and an update is required. In 2022, Waddell *et. al.* reported on the age, sex, and race-varying rates of cannabis use in veterans and non-veterans^11^. Although the demographic characteristics of these populations were assessed, this work focused on interactions of demographic details and veteran status as risk factors for alcohol and cannabis use^11^.

It is well known that cannabis pharmacokinetics and pharmacodynamics vary intra- and inter-personally^12^. Cannabis effects may be moderated by a wide range of factors such as sex, age, race, and ethnicity. For example, preclinical work from rodent ∆^9^-tetrahydrocannabinol (∆^9^-THC) dosing studies demonstrates that metabolism and bioaccumulation of ∆^9^-THC and psychoactive metabolites are significantly impacted by rodent sex. Human studies have also identified sex differences in subjective cannabis effects^13–16^, although additional exploration is needed. Similarly, aging is associated with metabolic changes, morbidities, and an overall decline in functioning^17^, likely impacting cannabis pharmacology. However, only a few studies have evaluated the pharmacology of cannabis in older adults^18–21^. The authors were unable to find literature describing demographics of people who currently or have recently used cannabis. However, two recent reviews and meta-analysis of published works on cannabis use disorder and behavioral health found that approximately 70% of study participants were male, 72% were non-Hispanic White, and the median participant age (SD) was 29.9 (9)^22–24^. However, balanced clinical trial participant pools must be demographically representative of those who use cannabis to gather generalizable results translatable to public policy. We hypothesize that cannabis clinical trial participants do not represent the sex, race, ethnicity, and age characteristics of people who use cannabis. One may argue that most fundamental pharmacokinetics and pharmacodynamics assessments of cannabis or cannabinoids do not aim to inform statutory or policy language. However, the lack of knowledge surrounding cannabis pharmacokinetics and impairment forces policy makers, enforcement officials, and other stakeholders to apply any available works to their immediate public health and safety needs. That is, results from any cannabis pharmacokinetics or pharmacodynamics studies in humans are likely to be read by and applied to those tasked with crafting evidence-based policies, recommendations, or assessments. This begs the question, what are the demographics of cannabis clinical trial participants, and do they reflect those of people who use cannabis? To begin answering this question, we will consider two data sources: (1) participant demographics extracted from a systematic review of cannabis pharmacokinetics and/or pharmacodynamics studies and (2) results from the United States National Survey on Drug Use and Health (NSDUH) from years 2002–2021. To the best of our knowledge, this is the first study comparing the demographics of cannabis clinical trial participants to those of people who use cannabis.

## Methods

### NSDUH survey results

The National Survey on Drug Use and Health (NSDUH) is a nationally representative and cross-sectional survey of individuals aged 12 years or older living in households or non-institutional group housing (e.g., college dormitories, but not jails or prisons) or with no permanent housing (e.g., residence in a shelter). The NSDUH uses a multistage area probability sample for each US state and the District of Columbia and an audio computer-assisted interviewing method to support confidential and private responses^25^. It is a key source of national and state-level data on the prevalence of substance use and health in the US.

Self-reported “past-month” cannabis use was examined by demographic characteristics within the 2002–2021 NSDUH data. All analyses used the Substance Abuse & Mental Health Data Archive (SAMHDA) Public-use Data Analysis System (PDAS) to query the NSDUH data. Self-reported “past-month” cannabis use (MRJMON, Rc-Marijuana—Past Month Use) was considered as a function of respondent sex (IRSEX, Imputed Revised Gender). Similarly, other variables were considered such as reported race and ethnicity (NEWRACE2, Rc-Race/Hispanicity Recode, 7 Levels). This included the following options: non-Hispanic White, non-Hispanic Black/African American, non-Hispanic Native American or Alaskan Native, non-Hispanic Native Hawaiian or Other Pacific Islander, non-Hispanic Asian, non-Hispanic more than one race, or Hispanic. A combined sex by race variable was also used (SEXRACE, Rc-Combined Gender by Race Indicator) for those who identify as non-Hispanic White, non-Hispanic Black, or Hispanic. Similarly, age was considered (CATAG6, Rc-Age Category Recode, 6 Levels) in the ranges of 12–17, 18–25, 26–34, 35–49, 50–64, and 65 + years old. Prior to 2005, the 6-Level age category was not available. Therefore, CATAG5 (Rc-Age Category Recode, 5 Levels) was used for years 2002 through 2004 for the age ranges of 12–17, 18–25, 26–34, and 35–49 years old. A combination of sex and age category was also used (SEXAGE, Rc-Combined Gender by Age Category Indicator) which identified the distribution of males and females within 12–17 and 18–25 age groups.

“Past-month” cannabis use was reported in eSupplement Table S1 as the weighted count, prevalence, and distribution within a population subgroup. Prevalence and distribution estimates were reported alongside their 95% confidence interval (CI). The prevalence was found by dividing the weighted count of “past-month” cannabis use by the weighted count of the population subgroup surveyed. For example, an estimated 12,861,131 non-Hispanic White males engaged in “past-month” cannabis use out of an estimated total of 84,158,445 non-Hispanic White males in the US. Therefore, it is estimated that 15.3% of non-Hispanic White males engaged in “past-month” cannabis use. The distribution was found by dividing the weighted count of “past-month” cannabis use by the weighted count of all who engaged in “past-month” cannabis use. For example, an estimated 12,861,131 non-Hispanic White males used cannabis in the “past-month” out of an estimated total of 36,172,820 people engaging in “past-month” cannabis use. This means non-Hispanic White males represent 35.6% of all people estimated to engage in “past-month” cannabis use.

Our study was exempt from IRB approval per the University of Wisconsin-Madison’s policy on publicly available, de-identified data sets. We followed the Strengthening the Reporting of Observational Studies in Epidemiology (STROBE) reporting guidelines for cross-sectional studies (e.g., clear variable specification, description of statistical analysis, and reporting 95% confidence intervals)^26^.

### Statistical analysis

Weighted crosstab analysis was used to identify and extract self-reported “past-month” cannabis use by age, sex, and race and ethnicity from 2002 through 2021. Results were displayed as weighted count, prevalence, and distribution (see eSupplement Table S1). Prevalence and distribution estimates also included 95% confidence intervals (CI). Time trends in “past-month” use prevalence across relevant population subgroups were calculated for 2002 through 2021. Logistic regression analysis was performed on annual prevalence estimates to identify statistically significant trend directionality (i.e., increase, decrease, no change) over time using R version 4.2.3 for Windows^27^. Regression lines were fitted to prevalence estimates using linear regression fits in which the dependent variable was the annual prevalence estimates. Linear regression models include slope (β), y-intercept (α), and their standard errors. Results from linear regression models are included as eSupplement Table S2. Goodness of fit was assessed through the residual standard error and coefficient of determination (R^2^) values. Residual standard error was found as the standard deviation of residuals using 18 degrees of freedom. A right-tailed F-test was used to identify model significance through the F-statistic (F) and p-value (p). Due to methodological changes in the 2005 NSDUH, the 6-level age category was not available for the 50–64 and 65 and older age ranges in years 2002 through 2004.

### Literature search strategy and study eligibility

Literature was identified that described the pharmacokinetics and/or pharmacodynamics of cannabis or cannabinoids in humans. Search terms included “cannabis”, “cannabinoids”, “cannabidiol”, “CBD”, “tetrahydrocannabinol”, “THC”, “pharmacokinetics”, “PK”, “pharmacodynamics”, and “PD” alone and in combination with one another. The search focused on literature presenting relevant models derived from data on humans published between January of 2000 and May of 2022. PubMed and Web of Science were searched and, following guidance from the Preferred Reporting Items for Systematic Reviews and Meta-analyses (PRISMA)^28,29^. This review was not registered with any databases and no protocol was prepared prior to literature search. The reference list was screened by both authors for inclusion in this work. After removal of duplicates, article titles and abstracts were considered by at least one author. Inclusion criteria included literature subjected to peer-review, data originating from a clinical trial, and description of a pharmacokinetics and/or pharmacodynamics model of one or more cannabinoids. Exclusion criteria included pre-clinical works, reviews, meta-analyses, novel drug delivery investigations, and efficacy-focused studies. Each full-text article was then assessed by both authors for inclusion in this work. Studies that re-analyzed data from previously published works or had clinical trials that generated multiple publications were coalesced.

### Data extraction

Both authors independently extracted the following data from each article: (1) participant type (i.e. healthy vs. patient), (2) previous cannabis exposure (i.e., naïve, mild, severe), (3) total number of participants, (4) administration route, (5) time of last plasma sample, (6) pharmacokinetics model (yes/no), (7) pharmacodynamics model (yes/no), (8) pharmacodynamics indicator reported, (9) participant sex, (10) participant age, and (11) participant race and ethnicity. Not all articles reported participant sex, age, race, and ethnicity.

## Results

### Shifting trends in recent cannabis use demographics

**Figure 1:**
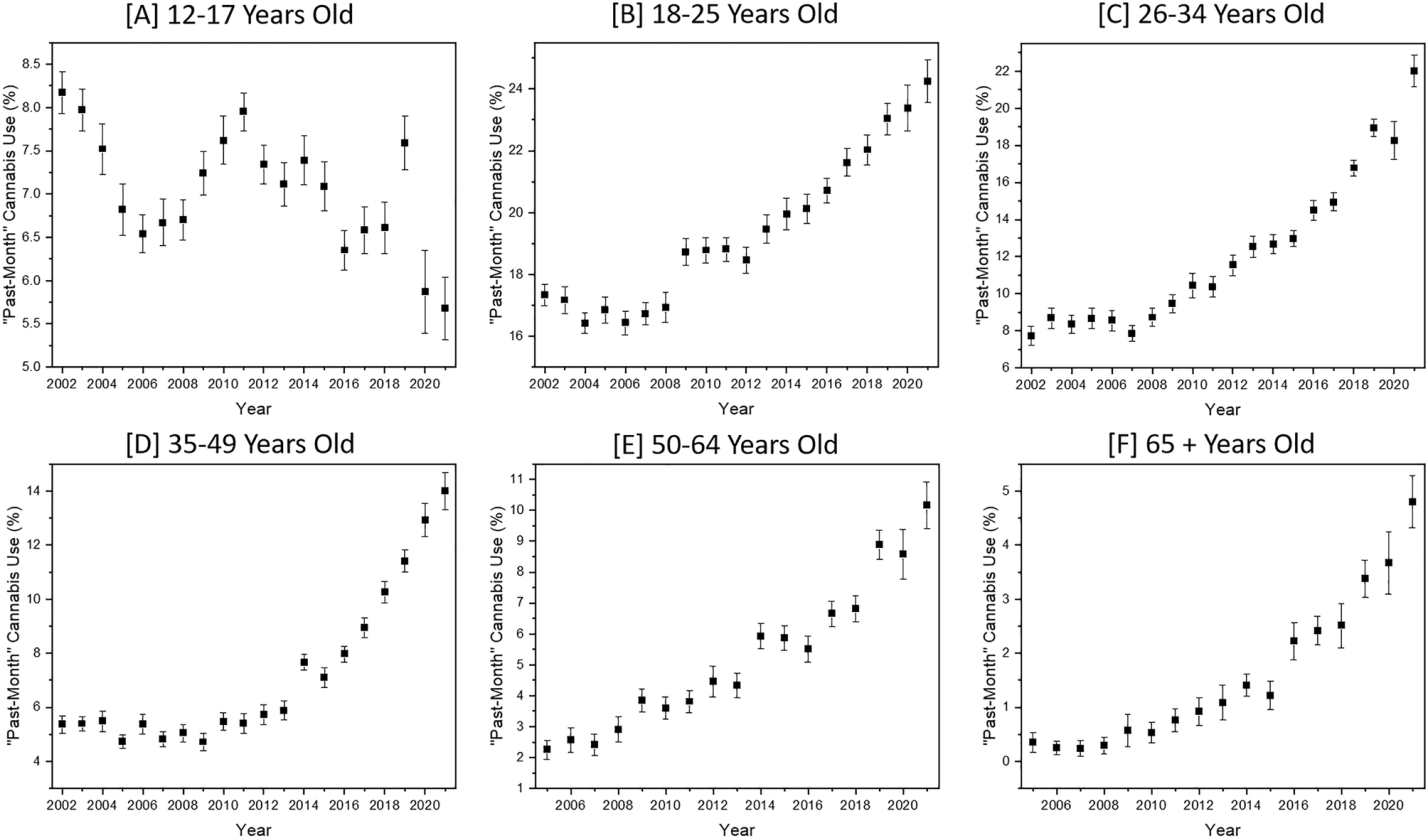
NSDUH data including the percentage of each group engaging in “past-month” cannabis use (prevalence) from years 2002 through 2021 for ages (**A**) 12–17, (**B**) 18–25, (**C**) 26–34, (**D**) 35–49, and from years 2005 through 2021 for ages (**E**) 50–64, and (**F**) 65 + years old.

In 2021, NSDUH estimates 12.9% of Americans (representing an estimated 36,172,820 people) used cannabis in the “past-month”^30^. This striking proportion of the population using cannabis recently and/or regularly is the culmination of a steady increase in societal acceptance of cannabis and cannabis use. Linear regression analysis identified trend directionality, and detailed results are provided in eSupplement Table S2. All population subgroups experienced significant changes in “past-month” cannabis use prevalence. Increases were identified in all age groups, Fig. 1, except 12–17-year-olds which decreased. The weighted counts of “past-month” cannabis use among 12–17-year-olds decreased 26.9% from 2002 to 2021 whereas 18–25-year-olds increased 50.9%. Similarly, the increase for 26–34-year-olds was 224.9% and 35–49-year-olds was 148.0%. Older Americans saw larger increases with 50–64-year-olds increasing 472.4% and those aged 65 and older increasing 2,066.1% from 2005 to 2021.

**Figure 2:**
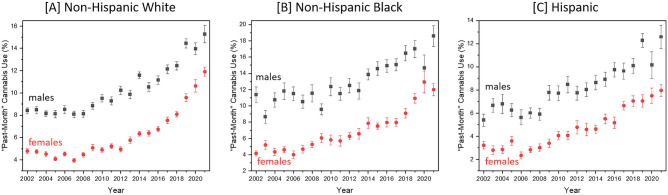
NSDUH data including the percentage of each group engaging in “past-month” cannabis use (prevalence) for years 2002 through 2021 for (**A**) non-Hispanic White males (black squares) and females (red circles), (**B**) non-Hispanic Black males (black squares) and females (red circles), and (**C**) Hispanic males (black squares) and females (red circles).

Increasing cannabis use is also observed, although unevenly, across sex and racial and ethnic groups, Fig. 2. Weighted counts of “past-month” cannabis use increased in non-Hispanic White males by 90.9%, non-Hispanic Black males by 115.8%, and Hispanic males by 292.7% from 2002 to 2021. Estimated increases in “past-month” cannabis use were larger for females as non-Hispanic White females increased by 153.4%, non-Hispanic black females by 258.6%, and Hispanic females by 332.4% from 2002 to 2021. These disproportionate increases in “past-month” cannabis use by females are closing the long-standing cannabis use sex gap. In 2021, 43.4% of people reporting “past-month” cannabis use were female.

### Recent cannabis use demographics

The demographics of people who have recently used cannabis was identified from 2021 NSDUH data. In 2021, a total of 69,850 US residents completed the NSDUH. Weighted demographics of respondents, as shown in eSupplement Table S3, were 48.9% male, 61.1% non-Hispanic White, 17.8% Hispanic, 12.2% non-Hispanic Black, and 5.8% non-Hispanic Asian. Due to the SARS-CoV-2 pandemic, NSDUH introduced web-based interviewing and the total number of completed interviews in 2020 was about half of prior years. A smaller sample size in 2020 impacts estimates for small population subgroups and rare behaviors. While the total number of completed interviews in 2021 returned to the annual goal of nearly 70,000, web-based interviewing persisted. For this work, 2020 response rates were adequate to generate national estimates of “past-month” cannabis use across the demographic variables considered. Therefore trend analysis included 2020 and 2021 data.

**Figure 3:**
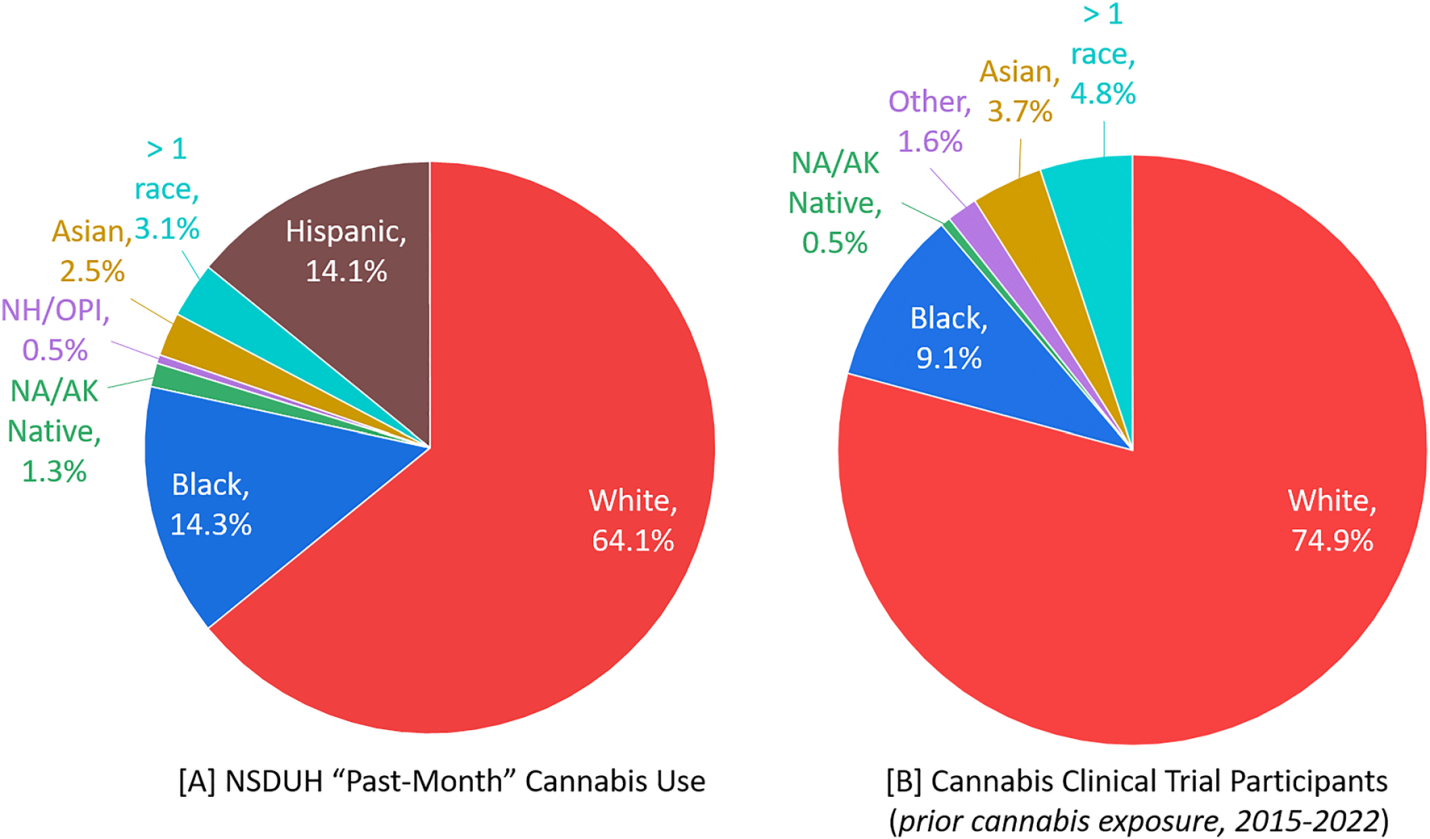
Comparison of the race and ethnicity of (**A**) people reporting “past-month” cannabis use via the NSDUH and (**B**) cannabis clinical trials including healthy participants with prior (mild or severe) cannabis experience which were published from 2015 to 2022. Herein Black represents Black or African American, NA/AK represents Native American or Alaskan Native, NH/OPI represents Native Hawaiian or Other Pacific Islander, and > 1 race represents two or more races. It must be noted that the NSDUH requires respondents to select their race and ethnicity simultaneously whereas clinical trial studies that reported these details treated race and ethnicity as separate, overlapping entities.

Overall, 12.9% of the 2021 respondents reported “past-month” cannabis use. Considering all people engaging in “past-month” cannabis use, demographic distributions were 43.4% female, 64.1% White, 14.3% Black or African American, 1.3% Native American or Alaskan Native, 2.5% Asian, 3.2% two or more races, and 14.1% Hispanic (see Fig. 3A). Those identifying as non-Hispanic White, non-Hispanic Black, and Hispanic were further broken down into male and female population subgroups, as shown in eSupplement Table S1. Within these population subgroups, males continue to report “past-month” cannabis use more often than females, although the gap has been shrinking in recent years (see Fig. 2). Considering age, the distribution of “past-month” cannabis use was predominantly in the 18–25 (22.4%), 26–34 (24.4%), 35–49 (24.1%), and 50–64 (17.6%) age ranges. However, as Fig. 1F shows, the prevalence of “past-month” cannabis use within the 65 years of age and older category is steadily increasing.

### Cannabis clinical trial participant demographics

**Figure 4:**
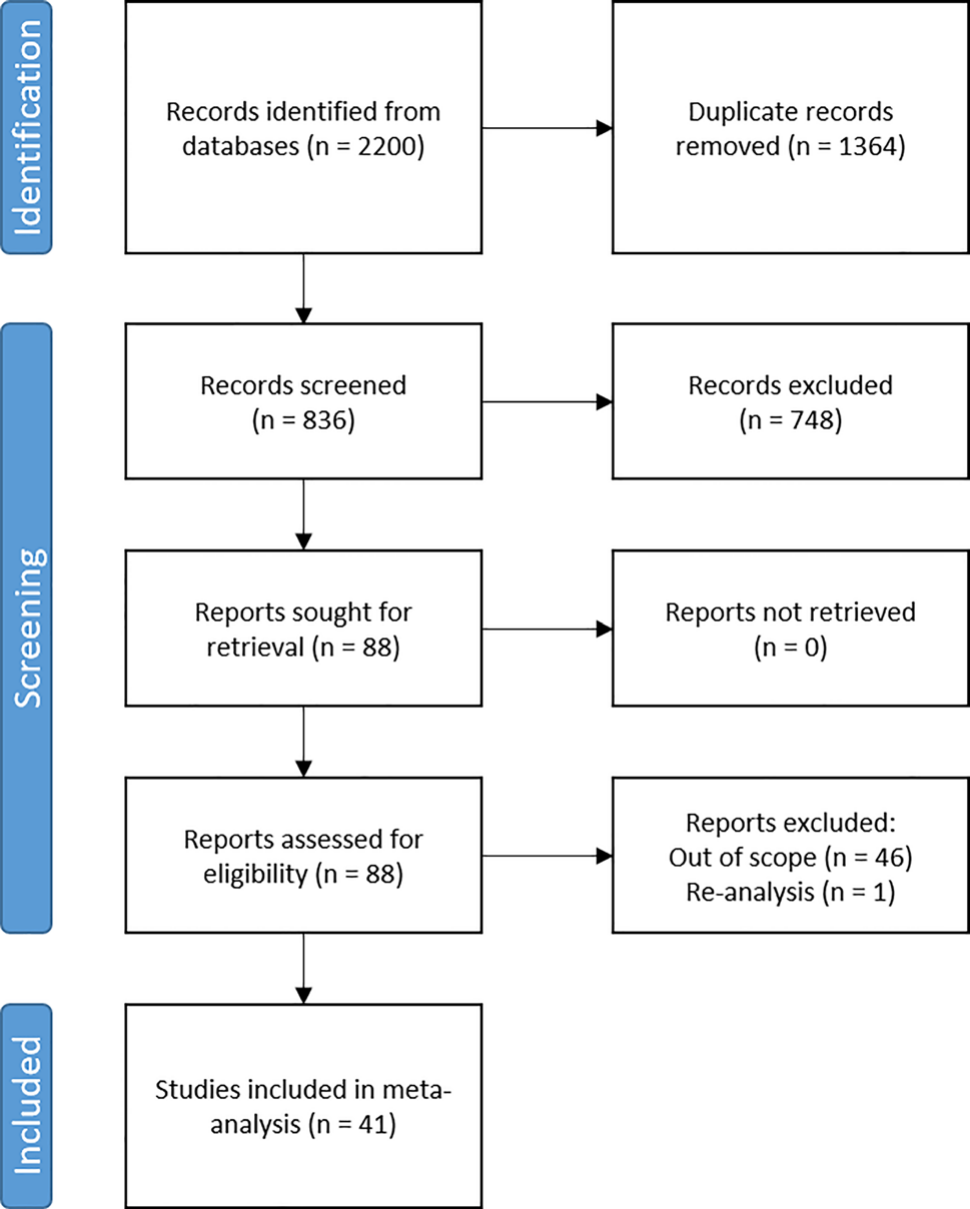
PRISMA 2020 flow diagram^29^.

Results of the literature search and selection process are detailed in Fig. 4. A total of 41 publications^16,31–70^ fit the search criteria and included unique participant pools, totaling 967 participants, see details in Table 1. Of those, 39 publications^16,31–43,45–63,65–70^ including 847 participants reported the number of males (573, 67.7%) and females (274, 32.3%). Only 20 publications^16,32,37,40–42,45,46,48,50,51,58–63,65,66,70^ reported at least some participant race information, see eSupplement Table S4. These publications included 404 (47.7% of total) participants, but race information was reported for 377 (93.3%) of those participants. From available information, participants were White (313, 83.0%), Black (24, 6.4%), Asian (9, 2.4%), two or more races (12, 3.2%), Native American (2, 0.5%), or "other" (3, 0.8%). Participant ethnicity was reported in 5 publications^45,48,60,65,70^ (11.9%), including 139 (16.4%) total participants and 14 (10.1%) who identified as Hispanic.

**Table 1:** Literature search results summary including article identifiers (first author, year published, and citation), type of participant and prior cannabis exposure (i.e., healthy vs. patient and naïve, mild, or severe, respectively), and number of participants (N). Here PK is pharmacokinetics and PD is pharmacodynamics. Publication year, patient participants, and those with no prior cannabis exposure are indicated in bold font as these characteristics were used to create literature subgroups within this work.

Article	Participant type	N	Route of administration	Last plasma sample (h)	PK model?	PD model?	PD indicator reported	N, Male	N, Female	Age range	Other participant details
Huestis, **2004**^31^	Healthy mild cannabis exposure	6	Smoking	24	Yes	No	–	6	0	Unreported	None provided
Guy, **2004**^32^	Healthy mild cannabis exposure	12	Oral	12	Yes	No	–	6	6	21–48	11 White, 1 two or more races, varied in height and weight
Guy, **2004**^33^	Healthy mild cannabis exposure	24	Oral	24	Yes	Yes	"High effect", questionnaire	24	0	18–50	None provided
Naef, **2004**^34^	Healthy **no prior cannabis exposure**	8	IV, inhalation	8	Yes	Yes	Pain test, questionnaire	4	4	26–50	Women: age range 26–35, body weight 60 ± 8 kg. Men: age range 27–50, body weight 80 ± 5 kg
Nadulski, **2005**^35^	Healthy mild cannabis exposure	24	Oral	24	Yes	No	–	12	12	18–45	Corrections for weight were applied to the women of this study
Strougo, **2008**^36^	Healthy mild cannabis exposure	12	Vaporized	10	Yes	Yes	"High" effect, heart rate, alertness	12	0	Unreported	None provided
Cooper, **2009**^37^	Healthy severe cannabis exposure	24	Smoking	3	Yes	Yes	"High effect", questionnaire, heart rate, CO levels	12	12	23–29	Women: 6 Black, 4 Hispanic, 2 two or more races/other, 25 ± 1 yrs. Men: 8 Black, 1 Hispanic, 1 White, 2 two or more races/other, 26 ± 3 yrs
Hunault, **2010**^38^	Healthy mild cannabis exposure	24	Smoking	8	Yes	No	–	24	0	18–33	BMI ranged from 20–25
Brenneisen, **2010**^39^	Healthy mild cannabis exposure	12	Smoking	8	Yes	Yes	"Hight effect", heart rate, blood pressure	12	0	23–29	BMI: 24 ± 2 kg/m^2^
Schoedel, **2011**^40^	Healthy severe cannabis exposure	23	Oral (spray/capsule)	8.5	Yes	Yes	Everything	Unreported	Unreported	20–45	83.3% male, 63.3% White, 23.3% Black
Karschner, **2011**^41^	Healthy severe cannabis exposure	9	Oral	10.5	Yes	No	–	6	3	19–43	(1) M, 19 yrs, White, 70 kg, 1.75 m. (2) F, 25 yrs, White, 66 kg, 1.73 m. (3) M, 22 yrs, Black, 95 kg, 1.78 m. (4) M, 28 yrs, Black, 64 kg, 1.68 m. (5) M, 27 yrs, Black, 73 kg, 1.78 m. (6) M, 43 yrs, Black, 109 kg, 1.96 m. (7) F, 20 yrs, Black, 69 kg, 1.45 m. (8) F, 21 yrs, White, 64 kg, 1.65 m. (9) M, 23 yrs, Black, 64 kg, 1.57 m
Joerger, **2012**^42^	**Patients** (amyotropic lateral sclerosis)	9	Oral	8	Yes	Yes	Heart rate	7	2	42–73	All White, body mass ranged from 59–84 kg
Lee, **2015**^43^	Healthy severe cannabis exposure	11	Smoking	Days later	Yes	No	–	10	1	25–52	None provided
Heuberger, **2015**^44^	Healthy mild, moderate, and severe cannabis exposure	84	IV, inhalation, oral	48	Yes	No	–	Unreported	Unreported	18–35	Average BMI ~ 22 kg/m^2^, comprised of 4 different datasets
Vandrey, **2017**^45^	Healthy mild cannabis exposure	18	Oral	130	Yes	Yes	"High effect", heart rate, questionnaire	9	9	20–33	13 White/Hispanic, 1 Black/non-Hispanic, 1 Asian/non-Hispanic, 1 American Indian/Hispanic
Marsot, **2017**^46^	Healthy mild cannabis exposure	12	Smoking	72	Yes	No	–	12	0	20–28	All White, "ideal" body weight
van Amerongen, **2018**^47^	**Patients** (multiple sclerosis)	24	Oral	Unreported	Yes	No	Pain, muscle spasticity	8	16	38–73	None provided
Schoedel, **2018**^48^	Healthy severe cannabis exposure	43	Oral	24	Yes	Yes	"High effect", heart rate, questionnaire	31	12	29–47	31 White, 8 Black, 3 Asian, 1 two or more races (of this set, 4 Hispanic and 39 non-Hispanic)
Awasthi, **2018**^49^	Healthy **no prior cannabis exposure**	8	IV	8	Yes	Yes	"High" effect	4	4	26–50	None provided
Birnbaum, **2019**^50^	**Patients** (refractory epilepsy)	8	Oral	72	Yes	Yes	Neurophyscological battery	6	2	27–79	7 White, 1 Black
Wolowich, **2019**^51^	Healthy **no prior cannabis exposure**	18	IV, oral	48	Yes	No	–	9	9	20–33	All White
Brands, **2019**^52^	Healthy severe cannabis exposure	91	Smoking	6	Yes	Yes	Driving capabilities, heart rate	64	27	20–25	None provided
Sempio, **2020**^53^	Healthy severe cannabis exposure	6	Smoking	168	Yes	No	–	6	0	29–36	Weight range: 64.8–93.4 kg
Liu, **2020**^54^	Healthy mild and severe cannabis exposure	36	Vaporized	1	Yes	No	–	31	5	19–51	Includes education (in years), intelligent quotient, along with weight and height info
Almog, **2020**^55^	**Patients** (chronic pain)	27	Inhalation	2.5	Yes	Yes	Pain intensity, vital signs, cognitive functions	19	8	18–69	None provided
Alvarez, **2020**^56^	Healthy moderate and severe cannabis exposure	30	Smoking	24	Yes	No	–	30	0	18–25	Education information is include: 76.7% university/further education, 13.3% high school, 10% vocational
Spindle, **2020**^57^	Healthy mild cannabis exposure	17	Oral	8	Yes	No	–	9	8	20–33	11 White, unknown for the other participants
Schuster, **2020**^58^	Healthy adolescent cannabis exposure	70	Unknown	*Urine: 28 days	Yes	No	–	44	26	15–25	68.6% White, 11.4% Black, 4.3% other, 11.4% two or more races, 4.3% Asian, 10% Hispanic
Sholler, **2020**^16^	Healthy mild cannabis exposure	50	Vaporized, oral	8	Yes	Yes	"High effect", questionnaire, vital signs	27	23	19–43	Weight range: 103–236 lbs
Spindle, **2020**^59^	Healthy mild cannabis exposure	18	Inhalation, oral	8	Yes	Yes	"High effect", questionnaire, cognitive and psychomotor battery	9	9	25–37	67% White/non-Hispanic, unknown for the other participants
Schlienz, **2020**^60^	Healthy mild cannabis exposure	17	Oral	8	Yes	Yes	"High effect", questionnaire, cognitive task performance	9	8	20–30	64.7% White, 17.6% Hispanic 94% with some college education
Hosseini, **2020**^61^	Healthy **no prior cannabis exposure**	12	Oral (spray/wafer/oil)	24	Yes	No	–	11	1	32–49	2 Asian, 10 White
Crockett, **2020**^62^	Healthy **no prior cannabis exposure**	30	Oral	96	Yes	No	–	12	18	22–50	26 White, 1 American Indian, 3 two or more races
Dahlgren, **2021**^63^	Healthy **no prior cannabis exposure**	14	Oral	*Urine	No	No	–	3	11	20–60	12 White, 2 Black, education years included as well (12–18)
Sempio, **2021**^64^	Healthy severe cannabis exposure	36	Smoking	1	Yes	Yes	Questionnaire	Unreported	Unreported	Unreported	None provided
Peters, **2021**^65^	Healthy **no prior cannabis exposure**	43	Oral	144	yes	yes	Questionnaire	21	22	21–33	86.0% White, 95.3% non-Hispanic
Schultz, **2022**^66^	Healthy **no prior cannabis exposure**	12	Oral	24	Yes	No	–	11	1	26–40	84.6% White, rest unknown
Sempio, **2022**^67^	Healthy severe cannabis exposure	6	Smoking	*Urine	Yes	No	–	6	0	Unreported	Described as "demographically similar"
Lorenzl, **2022**^68^	Healthy **no prior cannabis exposure**	12	Oral (spray)	30	Yes	Yes	Questionnaire	12	0	38–54	None provided
Bonomo, **2022**^69^	**Patients** (chronic non-cancer pain)	9	Oral	8	Yes	Yes	Pain intensity	7	2	52–64	None provided
Sholler, **2022**^70^	Healthy **no prior cannabis exposure**	18	Vaporized, oral	*Urine	Yes	No	–	9	9	21–41	14 White, 4 Black, 2 Hispanic

Detailed participant age information was available in 6 publications^38,41,43,55,58,63^ including 155 participants. Those works included participants in the following age ranges: 14–15 (1, 0.6%), 16–17 (1, 1.3%), 18–19 (25, 16.1%), 20–29 (79, 51%), 30–39 (19, 12.3%), 40–49 (10, 6.5%), 50–59 (9, 5.8%), and 60–69 (10, 6.5%). In addition, 37 publications^16,32–35,37–63,65,66,68–70^ reported age ranges including 907 participants. The minimum age considered was 15 and maximum was 79 years old. Considering the minimum age, the mean was 23.9, the median was 21, and the range was 15–52 years old. Considering the maximum age, the mean was 44.3, the median was 43, and the range was 25–79 years old.

#### Participant demographics from cannabis clinical trials published in 2000–2014 versus 2015–2022

To assess for shifts in cannabis clinical trial participant demographics over time, the above described data was partitioned into works published in 2000–2014 and those published in 2015–2022. Summaries of participant demographic characteristics for each literature analysis subgroup are included in eSupplement Table S4. A total of 12 manuscripts^31–42^ were published in 2000–2014 that met the inclusion criteria of this work. These studies included 187 participants of which most (144, 77.0%) were male. When race was reported, 100.0% of participants were White. Conversely, 29 manuscripts^16,43–70^ were published in 2015–2022 and met the inclusion criteria of this work. These studies included 780 participants of which 429 (65.0%) were male. Race was reported for 338 participants, including While (274, 81.1%), Black (24, 7.1%), Asian (9, 2.7%) Asian, two or more races (12, 3.6%), Native American (2, 0.6%), or "other" (3, 0.9%) participants. In this cohort (2015–2022), ethnicity was reported for 139 participants, with 14 (10.1%) identifying as Hispanic. Minimum and maximum age ranges were similar, when reported, for these works.

#### Participant demographics from cannabis clinical trials including only healthy participants with prior cannabis exposure

When applying cannabis clinical trial results to people who use cannabis, readers must consider if the clinical trial participants were patients or healthy and if they have prior experience with cannabis. To that end, we also considered cannabis clinical trial participant demographic characteristics (published in 2000-2022) when all “patients” and “no prior cannabis exposure” participants were removed, see eSupplement Table S4. This subgroup, called herein “prior cannabis exposure” included 26 publications^16,31–33,35–41,43–46,48,52–54,56–60,64,67^ including 715 participants of which 430 (72.3%) were male. Race was reported for 240 of these participants, including White (170, 78.3%), Black (17, 7.8%), Asian (7, 3.2%), two or more races (9, 4.1%), Native American (1, 0.5%), or other (3, 1.4%). Ethnicity was reported for 78 participants and 10 (12.8%) were Hispanic.

#### Participant demographics from cannabis clinical trials including only healthy participants with prior cannabis exposure published in 2015–2022

Within the above described “prior cannabis exposure” cohort, 10 manuscripts were published between 2000 and 2014. Removing those, a final subgroup was created including “prior cannabis exposure” participants that were published between 2015 and 2022, see eSupplement Table S4. This group included 16 works^16,43–46,48,52–54,56–60,64,67^ and 545 participants that should be most similar to the demographic characteristics of NSDUH “past-month” cannabis use survey results. Within this group, 297 (69.9%) participants were male. Race was reported for 187 participants including those who identified as White (140, 74.9%), Black (17, 9.1%), Asian (7, 3.7%), two or more races (9, 4.8%), Native American (1, 0.5%), or "other" (3, 1.6%). The distribution of racial groups (when available) included in cannabis clinical trials reporting pharmacokinetics and/or pharmacodynamics models are shown in Fig. 3B. It must be noted that the NSDUH requires respondents to select their race and ethnicity simultaneously whereas clinical trial studies that reported these details treated race and ethnicity as separate, overlapping entities. Therefore, Hispanic ethnicity totals were not included in Fig. 3B as only 3 publications^45,48,60^ reported the participant’s ethnicity, including 78 total participants and 10 (12.8%) who identified as Hispanic. Participant ages were primarily reported as minimum to maximum age ranges. The range of minimum ages was 15–29 years old, with an average minimum age of 21.2 years old. The range of maximum ages was 25–52 years old, with an average maximum age of 35.7 years old.

## Discussion

This is the first study to compare the NSDUH demographics of people reporting “past-month” cannabis use to cannabis clinical trial participants. The main finding of this study is that demographics of those reporting “past-month” cannabis use are quite diverse, with significant prevalence increases between 2002 and 2021 in older Americans, women, and historically underrepresented groups. However, cannabis clinical trial participants continue to be majority White males in their 20s and 30s. Literature published recently (2015–2022) have included more women and racial minorities compared to literature from 2000–2014. However, this chasm between the demographics of people reporting recent cannabis use and clinical trial participants is concerning. It is well known that demographic differences are an important factor in interpersonal variability in the pharmacokinetics and pharmacodynamics of a substance^71^. Therefore, any pharmacokinetics or pharmacodynamics models generated from these cannabis clinical trials may not be generalizable to the increasingly diverse population of people who use cannabis.

Over the past 18 years, the prevalence of “past-month” cannabis use increased across all age categories except 12–17-year-olds. While decreasing prevalence of “past-month” cannabis use in the pediatric population is encouraging, significant increases in other age groups warrants increased awareness and scientific inquiry. Increases in “past-month” cannabis use prevalence was not evenly distributed across all age groups considered. For example, “past-month” cannabis use prevalence increased 50.9% in 18–25-year-olds from 2002 to 2021 whereas the increase was 224.9% in 26–34 and 148.0% in 35–49-year-olds. Increases were even larger for 50–64-year-olds at 472.4% and use by those aged 65 and older increased an astonishing 2,066.1%. Significant increases in “past-month” cannabis use by older Americans has not received a commiserate increase in research interest and clinical investigation^72–74^. In cannabis clinical trials, age ranges were reported for most participants (94.0%), but detailed age information down to the decade was only reported on some (15.6%) participants. Furthermore, removal of patient participant works reduced the overall age range of participants included in studies. Pharmacokinetics and pharmacodynamics clinical trials are inherently limited by small sample size and stringent inclusion and exclusion criteria. Despite these limitations, the rapid expansion of “past-month” cannabis use by adults aged 50 and older warrants additional clinical investigation.

To understand how cannabis clinical trial participant demographic characteristics have changed over time, data extracted from the literature was divided into several subgroups. These subgroups were: works published in 2000–2014, works published in 2015–2022, works including healthy participants with prior cannabis experience, and works including healthy participants with prior cannabis experience which were published from 2015 to 2022. With respect to publication date, promising improvements in participant demographic characteristics are apparent. Works published in the years 2015–2022 included more women and historically underrepresented racial and ethnic groups than those published in 2000–2014. Including participant eligibility criteria (i.e., healthy with prior cannabis experience) alongside publication years of 2015–2022 boosted the racial and ethnic diversity of participants, but decreased female representation. Furthermore, removal of patients (studies assessing cannabis for therapeutic applications) reduced the age range of included participants.

Cannabis clinical trial participant demographic information was significantly lacking in the works considered here. Participant sex was commonly (87.9%) reported, but race (39.1%) and ethnicity (14.0%) details were deficient. When participant demographic data is missing, the reader is unable to critically assess the translatability of reported results to “real world” cannabis use. Even when available, race and ethnicity data is oversimplified and incomplete. For example, NSDUH crudely aggregates all those identifying as Hispanic together, limiting our ability to consider how ethnicity overlaps with different racial groups in those who report “past-month” cannabis use. However, cannabis clinical trial participant demographics largely failed to capture any ethnicity information. This represents structural discrimination in the research landscape that perpetuates social and health inequities. Overall, there needs to be greater transparency and reporting of participant demographics.

This study had several limitations. Cannabis clinical trial participant data was derived from peer-reviewed literature, which introduces publication bias. That is, this work fails to capture any studies that were completed but not published, for whatever reason. Additionally, cannabis clinical trials often include intentionally stringent participant eligibility and ineligibility criteria. For example, females of reproductive potential may be ineligible due to teratogenicity risk. Additionally, the small sample sizes used in pharmacokinetics and pharmacodynamics studies may result in narrow eligible age ranges, preventing consideration of older adults. This is exemplified by studies on therapeutic potential of cannabis and cannabinoids (i.e., participants were patients) for certain indications which tended to include older adults.

## Conclusion

Significant investments have facilitated the wide availability of NSDUH results and datasets to the public through the Substance Abuse and Mental Health Data Archive (https://pdas.samhsa.gov/). Therefore, demographic details on people reporting cannabis use, including “past-month”, “past-year”, or “lifetime” cannabis use, is available. This data and online data exploration tool was chosen to promote its use when designing future cannabis clinical trials. As shown in this work, the demographics of people reporting “past-month” cannabis use has changed over time. These changes across age, sex, and race and ethnicity necessitate annual reconsideration of the demographics of people who use cannabis. However, cannabis clinical trials seeking to generate pharmacokinetics and/or pharmacodynamics models of cannabis or cannabinoids do not include participants whose demographics are representative of those who use cannabis. This disconnect is problematic for those seeking translatable data to inform public policy on cannabis use and cannabis products. Well-crafted clinical trials should consider the demographics described in this work and updated annually through the Substance Abuse and Mental Health Data Archive. Funding agencies should also consider this data when evaluating funding proposals and crafting Requests for Proposals and Notices of Special Interest announcements.

## Supplementary Information

Supplementary Tables.

## Data Availability

All data used in this work is publicly available through the Substance Abuse and Mental Health Data Archive (https://pdas.samhsa.gov/#/).
