# Supplementary material for: Measuring the diversity gap of cannabis clinical trial participants compared to people who report using cannabis
Source: Sci Rep. 2023 Jun 16;13:9787. doi: 10.1038/s41598-023-36770-5 (PMC10276002; doi:10.1038/s41598-023-36770-5)
Supplement: Supplementary file 1 — Supplementary Tables. [file 41598_2023_36770_MOESM1_ESM.docx]

**Measuring the diversity gap of cannabis clinical trial participants compared to people who report using cannabis**

**Heather Barkholtz**^1, 2,^ * and **Maia Bates** ^1, 3^

^1^Forensic Toxicology, Environmental Health Division, Wisconsin State Laboratory of Hygiene, 2601 Agriculture Dr., Madison, WI 53718, USA

^2^Pharmaceutical Sciences, School of Pharmacy, University of Wisconsin-Madison, 777 Highland Ave., Madison, WI 53705, USA

^3^Department of Chemistry, College of Letters of Science, University of Wisconsin-Madison, 1101 University Ave., Madison, WI 53706, USA

*Corresponding author: phone: (608) 890-1967, email: [hbarkholtz@wisc.edu](mailto:hbarkholtz@wisc.edu)

**Table S1**. Demographic characteristics of study population by cannabis use in the United States, 2021. Prevalence is the weighted count of “past-month” cannabis use divided by the weighted count of the population subgroup surveyed (displayed in parenthesis in the characteristic column). Distribution is the weighted count of “past-month” cannabis use divided by the weighted count of overall “past-month” cannabis use (here: 36,172,820). Prevalence and distribution estimates include 95% confidence intervals (CI).

| **Characteristic (Weighted total)** | **Past-month cannabis use** | | |
| --- | --- | --- | --- |
|  | **Weighted Count** | **Prevalence (CI), %** | **Distribution (CI), %** |
| Overall (279,843,944) | 36,172,820 | 12.93 (12.22-13.66) | --- |
| Male (136,763,692) | 20,471,904 | 14.97 (13.84-16.17) | 56.59 (54.52-58.64) |
| Female (143,080,251) | 15,700,917 | 10.97 (10.34-11.64) | 43.41 (41.36-45.48) |
| **Race/ethnicity** | | | |
| White (171,099,411) | 23,206,004 | 13.56 (12.66-14.52) | 64.15 (61.85-66.39) |
| Male (84,158,445) | 12,861,131 | 15.28 (13.75-16.95) | 35.55 (33.05-38.14) |
| Female (86,940,967) | 10,344,873 | 11.90 (11.10-12.74) | 28.60 (26.70-30.57) |
| Black (34,267,092) | 5,157,148 | 15.05 (13.57-16.66) | 14.26 (12.54-16.17) |
| Male (15,901,614) | 2,953,845 | 18.58 (16.16-21.26) | 8.17 (7.05-9.44) |
| Female (18,365,478) | 2,203,303 | 12.00 (10.59-13.56) | 6.09 (5.22-7.10) |
| Native American/Alaskan Native (1,772,091) | 486,494 | 27.45 (20.10-36.28) | 1.34 (0.94-1.91) |
| Native Hawaiian/Other Pacific Islander (1,099,568) | 175,609 | 15.97 (9.97-24.60) | 0.49 (0.29-0.82) |
| Asian (16,344,245) | 894,816 | 5.47 (4.18-7.14) | 2.47 (1.88-3.25) |
| More than one race (5,479,187) | 1,138,982 | 20.79 (17.40-24.64) | 3.15 (2.50-3.95) |
| Hispanic (49,782,349) | 5,113,767 | 10.27 (9.10-11.58) | 14.14 (12.59-15.84) |
| Male (24,942,345) | 3,136,974 | 12.58 (10.68-14.75) | 8.67 (7.40-10.14) |
| Female (24,840,005) | 1,976,793 | 7.96 (6.97-9.07) | 5.46 (4.82-6.19) |
| **Age Groups** | | | |
| 12-17 (26,019,281) | 1,478,621 | 5.68 (5.00-6.46) | 4.09 (3.55-4.71) |
| Male (13,288,095) | 683,384 | 5.14 (4.24-6.23) | 1.89 (1.55-2.30) |
| Female (12,731,187) | 795,237 | 6.25 (5.25-7.42) | 2.20 (1.78-2.71) |
| 18-25 (33,458,433) | 8,113,395 | 24.25 (22.88-25.67) | 22.43 (21.01-23.91) |
| Male (16,723,779) | 4,364,411 | 26.10 (23.89-28.43) | 12.07 (10.95-13.28) |
| Female (16,734,654) | 3,748,983 | 22.40 (20.83-24.05) | 10.36 (9.54-11.26) |
| 26-34 (40,161,932) | 8,841,625 | 22.01 (20.35-23.78) | 24.44 (22.58-26.41) |
| 35-49 (62,187,800) | 8,707,700 | 14.00 (12.70-15.42) | 24.07 (22.07-26.20) |
| 50-64 (62,856,466) | 6,385,496 | 10.16 (8.74-11.78) | 17.65 (15.43-20.12) |
| 65 + (55,160,031) | 2,645,983 | 4.80 (3.91-5.87) | 7.31 (6.01-8.88) |

**Table S2**. Linear regression analysis detailed results of cannabis use trends from 2002 through 2021. Residual standard error is the standard deviation of the residuals and R^2^ is the coefficient of determination of the linear regression. A right-tailed F-test yielded the F-statistic (F) and p-value (p). Linear regression models include slope (β), y-intercept (α), and their standard errors.

| **Population Subgroup** | **Residual standard error** | **R^2^** | **F** | **p** | **β** | **β standard error** | **α** | **α standard error** |
| --- | --- | --- | --- | --- | --- | --- | --- | --- |
| **Age Groups** | | | | | | | | |
| 12-17 | 0.5761 | 0.3165 | 8.337 | 0.009811 | -0.06450 | 0.02234 | 136.8 | 44.94 |
| 18-25 | 0.7955 | 0.9015 | 164.8 | < 0.001 | 0.3961 | 0.03085 | -777.3 | 62.06 |
| 26-34 | 1.475 | 0.8818 | 134.3 | < 0.001 | 0.6625 | 0.05718 | -1320 | 115.0 |
| 35-49 | 1.517 | 0.7337 | 49.60 | < 0.001 | 0.4143 | 0.05883 | -826.2 | 118.3 |
| 50-64 | 0.6644 | 0.9277 | 192.4 | < 0.001 | 0.4562 | 0.03289 | -913.2 | 66.21 |
| 65 + | 0.5253 | 0.8635 | 94.88 | < 0.001 | 0.2533 | 0.02601 | -508.4 | 52.35 |
| **Combined Race and Sex Groups** | | | | | | | | |
| White male | 0.885 | 0.8561 | 107.1 | < 0.001 | 0.3566 | 0.03446 | -706.9 | 69.31 |
| White female | 1.135 | 0.7616 | 57.39 | < 0.001 | 0.3335 | 0.04403 | -664.8 | 88.57 |
| Black male | 1.250 | 0.7801 | 63.85 | < 0.001 | 0.3874 | 0.04848 | -766.3 | 97.52 |
| Black female | 1.056 | 0.8444 | 97.68 | < 0.001 | 0.4046 | 0.04094 | -806.9 | 82.34 |
| Hispanic male | 0.8131 | 0.8549 | 106.0 | < 0.001 | 0.3246 | 0.03153 | -644.8 | 63.42 |
| Hispanic female | 0.6197 | 0.8828 | 135.5 | < 0.001 | 0.2798 | 0.02403 | -558.1 | 48.34 |

**Table S3**. NSDUH 2021 respondent demographics. Actual sample size is the raw number of completed interviews. Weighted sample and its standard error are population estimates.

| **Characteristic** | **Actual Sample Size** | **Weighted Sample** | **Weighted Sample Standard Error** |
| --- | --- | --- | --- |
| Total Responses | 69,850 | 279,843,944 | 5,072,751.7 |
| Male | 31,873 | 136,763,692 | 2,636,349.1 |
| Female | 37,977 | 143,080,251 | 2,791,278.8 |
| **Not Hispanic or Latino** | | | |
| White | 43,465 | 171,099,411 | 3,004,717.1 |
| Black or African American | 7,796 | 34,267,092 | 1,232,300.3 |
| Asian | 7,148 | 16,344,245 | 707,623.5 |
| AIAN |  | 1,772,091 | 185,383.0 |
| NHOPI |  | 1,099,568 | 183,154.9 |
| Two or More Races |  | 5,479,187 | 343,329.2 |
| Hispanic or Latino | 11,441 | 49,782,349 | 2,646,966.4 |

**Table S4**. Summary of clinical trial participant demographic characteristics by various literature groupings. All included works were divided into subgroups including: those published in 2000-2014, those published in 2015-2022, those including healthy participants with prior cannabis experience, and those including healthy participants with prior cannabis experience which were published from 2015-2022. Additionally, the summary of all works was included as 2000-2022 (*all works*) in bold for comparative purposes.

| **Participant Sex** | | | | | | | | | | | |
| --- | --- | --- | --- | --- | --- | --- | --- | --- | --- | --- | --- |
| **Condition** | **Total N** | | **Men (%)** | | | | **Women (%)** | | | | |
| **2000-2022 (*all works*)** | **967** | | **67.7** | | | | **32.3** | | | | |
| 2000-2014 | 187 | | 77.0 | | | | 23.0 | | | | |
| 2015-2022 | 780 | | 65.0 | | | | 35.0 | | | | |
| Prior cannabis exposure | 715 | | 72.3 | | | | 27.7 | | | | |
| Prior cannabis exposure + 2015-2022 | 545 | | 69.6 | | | | 30.1 | | | | |
| **Participant Race and Ethnicity** | | | | | | | | | | | |
| **Condition** | **Total N** | **Race Reported N** | **White (%)** | **Black (%)** | **Asian (%)** | | **> 1 race (%)** | **NA (%)** | | **Other (%)** | **Hispanic (%)** |
| **2000-2022 (*all works*)** | **967** | **377** | **83.0** | **6.4** | **2.4** | | **3.2** | **0.5** | | **0.8** | **3.7** |
| 2000-2014 | 187 | 39 | 100.0 | --- | --- | | --- | --- | | --- | --- |
| 2015-2022 | 780 | 338 | 81.1 | 7.1 | 2.7 | | 3.6 | 0.6 | | 0.9 | 4.1 |
| Prior cannabis exposure | 715 | 217 | 78.3 | 7.8 | 3.2 | | 4.1 | 0.5 | | 1.4 | 4.6 |
| Prior cannabis exposure, 2015-2022 | 545 | 187 | 74.9 | 9.1 | 3.7 | | 4.8 | 0.5 | | 1.6 | 5.3 |
| **Participant Age Ranges** | | | | | | | | | | | |
| **Condition** | **Total N** | **Minimum age range** | **Average minimum age** | | | **Maximum age range** | | | **Average maximum age** | | |
| **2000-2022 (*all works*)** | **967** | **15-52** | **23.9** | | | **25-79** | | | **44.3** | | |
| 2000-2014 | 187 | 18-42 | 22.8 | | | 29-73 | | | 44.5 | | |
| 2015-2022 | 780 | 15-52 | 24.4 | | | 25-79 | | | 44.2 | | |
| Prior cannabis exposure | 715 | 15-29 | 20.8 | | | 25-52 | | | 37.4 | | |
| Prior cannabis exposure + 2015-2022 | 545 | 15-29 | 21.2 | | | 25-52 | | | 35.7 | | |
